# RECQL4 is not critical for firing of human DNA replication origins

**DOI:** 10.1038/s41598-024-58404-0

**Published:** 2024-04-02

**Authors:** Laura Padayachy, Sotirios G. Ntallis, Thanos D. Halazonetis

**Affiliations:** Department of Molecular and Cellular Biology, University of Geneva, 1205 Geneva, Switzerland

**Keywords:** DNA replication, DNA replication

## Abstract

Human RECQL4, a member of the RecQ helicase family, plays a role in maintaining genomic stability, but its precise function remains unclear. The N-terminus of RECQL4 has similarity to Sld2, a protein required for the firing of DNA replication origins in budding yeast. Consistent with this sequence similarity, the *Xenopus laevis* homolog of RECQL4 has been implicated in initiating DNA replication in egg extracts. To determine whether human RECQL4 is required for firing of DNA replication origins, we generated cells in which both *RECQL4* alleles were targeted, resulting in either lack of protein expression (knock-out; KO) or expression of a full-length, mutant protein lacking helicase activity (helicase-dead; HD). Interestingly, both the *RECQL4* KO and HD cells were viable and exhibited essentially identical origin firing profiles as the parental cells. Analysis of the rate of fork progression revealed increased rates in the *RECQL4* KO cells, which might be indicative of decreased origin firing efficiency. Our results are consistent with human RECQL4 having a less critical role in firing of DNA replication origins, than its budding yeast homolog Sld2.

## Introduction

DNA replication is a highly regulated process leading to duplication of the genome. In eukaryotes, DNA replication entails licensing of DNA replication origins in the G1 phase of the cell cycle and then firing of these origins in S phase, resulting in the establishment of two replisomes per origin that copy DNA in a bidirectional manner. DNA replication is completed when replisomes from adjacent origins converge^1–3^. Origin licensing involves the loading of a minichromosome maintenance (MCM) double-hexamer on double-stranded DNA. During origin firing, additional proteins are recruited to the MCM double-hexamer, resulting in the separation of the two MCM hexamers from each other and melting of the double-stranded DNA, such that each MCM hexamer ends up encircling single-stranded DNA^4^.

Origin firing involves several steps, as first demonstrated in budding yeast. In the first step, the proteins Sld3 and Sld7 are recruited to the MCM double-hexamer, in a manner dependent upon phosphorylation of MCM subunits by the Dbf4-dependent kinase (DDK), bringing with them Cdc45 to the MCM^5–7^. A second step involves the formation of a preloading complex (pre-LC) containing Sld2, Dpb11, GINS and DNA polymerase epsilon^8^. The formation of the pre-LC is dependent upon cyclin-dependent kinase (CDK) activity^8^. CDK phosphorylates Sld2, leading to binding of Sld2 to Dpb11; CDK also phosphorylates Sld3, which then also interacts with Dpb11. These CDK-dependent interactions result in the recruitment of the pre-LC to the MCM-Sld3-Cdc45 complex^7–10^. In budding yeast, artificial formation of an Sld3-Dpb11-Sld2 complex bypasses the requirement of CDK for origin firing^9,10^.

Proteins with sequence similarity to budding yeast Sld3, Sld7 and Dpb11 have been identified in higher eukaryotes; in humans, they are called TRESLIN (encoded by the *TICRR* gene), MTBP and TOPBP1, respectively^11–15^. A TRESLIN-MTBP heterodimer recruits CDC45 to the MCM in a DDK-dependent manner; furthermore, TRESLIN is phosphorylated by CDK2-Cyclin E, leading to binding of TRESLIN to TOPBP1 and recruitment of TOPBP1 to the MCM^16–19^. Thus, the function of these proteins, which corresponds to the first step of origin firing in budding yeast, appears to be well-conserved in evolution.

The GINS protein complex and DNA polymerase epsilon, which are part of the pre-LC complex in budding yeast, are also well-conserved in higher eukaryotes. However, Sld2 is less well conserved; RECQL4, the homologous human protein, has an N-terminal domain with sequence similarity to Sld2, but also has a helicase domain that is absent in budding yeast Sld2^20–23^. The helicase domain places RECQL4 in the family of RECQ helicases, of which there are five members in humans. Each member of the RECQ helicase family has additional N- or C-terminal domains that are unique to that member, as well as somewhat different DNA helicase activities. RECQL4 has the most divergent helicase domain in the family and possesses DNA strand re-annealing activity, in addition to its helicase activity^23^.

Interestingly, the Sld2 residue, that is phosphorylated by CDK in budding yeast and is important for the interaction of Sld2 with Dpb11, is not conserved in human RECQL4^11^. Nevertheless, there is strong evidence that RECQL4 and, in particular, its N-terminal domain is important for initiation of DNA replication in higher eukaryotes, including *C. elegans*^24^, *Drosophila*^25–27^, *Xenopus*^20,21^ and vertebrates^28–30^. However, at least in *Xenopus*, it appears that RECQL4 acts at a step after the recruitment of GINS^20,21^, whereas, in budding yeast, Sld2 mediates the recruitment of GINS to the replisome^7–10^.

In this study, we investigated the role of human RECQL4 in initiation of DNA replication using *RECQL4* knock-out (KO) and helicase-dead (HD) knock-in cells. Analysis of DNA replication in these cells suggests that RECQL4 is not critical for origin firing, contrary to the essential function of Sld2 in budding yeast, but consistent with emerging evidence that there are substantial differences in the mechanism of origin firing between yeast and higher eukaryotes^31–36^.

## Results

### RECQL4 is not critical for initiation of DNA replication

**Figure 1:**
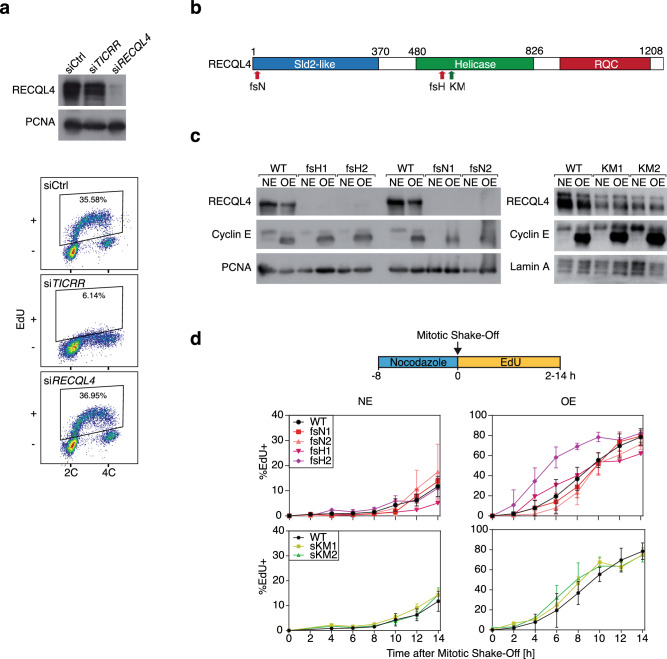
| RECQL4 is not critical for entry into S phase. (**a**) Immunoblot showing depletion of RECQL4 by specific siRNA (si) with PCNA serving as a loading control (top) and flow cytometry analysis of EdU incorporation in asynchronous cells following depletion of TRESLIN or RECQL4 (bottom). Ctrl, control; 2C and 4C, genomic DNA content. (**b**) Graphical representation of the human RECQL4 protein showing its three major protein domains and the sites targeted for mutagenesis. Red and green arrows, sites targeted in the KO and HD clones, respectively. fsH and fsN, frameshift mutations in the Helicase and N-terminal domains, respectively; KM, lysine to methionine substitution in the helicase domain. (**c**) Immunoblot analysis showing the levels of RECQL4 expression in the KO and HD clones and induction of Cyclin E. PCNA and Lamin A serve as loading controls. NE, normal levels of Cyclin E; OE, overexpression of Cyclin E. (**d**) Experimental setup (top) and kinetics of S-phase entry of parental cells and *RECQL4*-mutant clones, as ascertained by flow cytometry-based analysis of EdU-positive cells (% EdU +) at different time points after mitotic shake-off (bottom). Averages and standard deviations from three independent experiments are shown. Clone fsH2 entered S phase faster than the parental cells, when Cyclin E was overexpressed (*P* < 0.001).

As a first step to determine whether RECQL4 is required for DNA replication, we monitored the cell cycle profile of U2OS osteosarcoma cells after depleting RECQL4 by siRNA. As controls, we examined cells transfected with a non-specific siRNA or with siRNA targeting *TICCR*. Depletion of RECQL4 had no apparent effect on DNA replication, as ascertained by incorporation of 5-ethynyl-2’-deoxyuridine (EdU), a thymidine analogue, into genomic DNA. In contrast, depletion of TRESLIN, the protein product of the *TICCR* gene, suppressed almost completely EdU incorporation (Fig. 1a and Supplementary Fig. 1a).

Considering the possibility that low levels of RECQL4 might be sufficient for DNA replication, we utilized CRISPR/Cas9-guided mutagenesis to inactivate the *RECQL4* gene. This was done in the context of a U2OS cell line, in which overexpression of the oncogene *CCNE1*, encoding Cyclin E, is induced by a Tet-OFF system^37^. Inducible *CCNE1* overexpression allowed us to study the function of RECQL4 in the absence and presence of oncogene-induced DNA replication stress, which is known to affect origin firing^38^. We generated KO clones, in which either exon 1 or exon 9 of *RECQL4* were targeted; these exons encode part of the N-terminal Sld2-like domain and part of the helicase domain, respectively (Fig. 1b and Supplementary Fig. 1b). Two clones targeting exon 1 and two clones targeting exon 9 were further analyzed. All clones had small genomic deletions or insertions that affected both alleles, resulting in premature stop codons (Supplementary Fig. 2). We will refer to these clones as frameshift N-terminal domain (fsN) or frameshift helicase domain (fsH) and collectively as KO.

No full-length RECQL4 protein could be detected in the KO clones by immunoblotting with two different commercially-available antibodies (Fig. 1c and Supplementary Fig. 3a). Moreover, the clones did not express truncated RECQL4 proteins, corresponding to the N-terminal domain that has homology to yeast Sld2 (Supplementary Fig. 2d), as such proteins would have been detected by the antibodies that we used. Specifically, the NBP2-47310 antibody recognized ectopically expressed polypeptides corresponding to residues 1–321 and 1–371 of human RECQL4, as well as full-length human RECQL4 (Supplementary Fig. 3a, b, c). The absence of detectable RECQL4 protein in the fsN clones was expected, whereas for the fsH clones, the most likely explanation for lack of expression of truncated RECQL4 protein is degradation of the mRNA by nonsense-mediated decay^39^.

We also generated mutant clones harboring a lysine 508 to methionine substitution within the helicase domain (Fig. 1b and Supplementary Fig. 1b, c). This substitution disrupts the ATP-binding site rendering RECQL4 unable to hydrolyze ATP and unwind DNA^40,41^. Two HD clones, referred to as KM1 and KM2, were further analyzed and validated by sequencing (Supplementary Fig. 2c). Both were found to express full-length RECQL4 protein, albeit at somewhat lower levels than the wild-type protein in the parental cells (Fig. 1c).

The ability to obtain all the clones described above suggests that neither the Sld2-like N-terminal domain, nor helicase activity are required for DNA replication and cell proliferation. Nevertheless, a more careful analysis of DNA replication was clearly warranted and we started by examining the kinetics of S phase entry. Parental U2OS cells and the *RECQL4* mutant clones were synchronized by mitotic shake-off and released into G1; the fraction of cells entering S-phase was then monitored over time by labeling the cells with EdU. The experiment was performed with cells expressing either normal levels of Cyclin E (NE) or overexpressing Cyclin E (OE). Although there was variation in the length of the G1 phase among clones, with the exception of one clone that entered S phase faster than the parental cells, their kinetics of entry into S phase was similar to that of the parental cells (Fig. 1d). This was true even when Cyclin E was overexpressed and the length of the G1 phase was significantly shortened (Fig. 1d).

### DNA replication origin firing profiles are unaffected by inactivation of *RECQL4*

**Figure 2:**
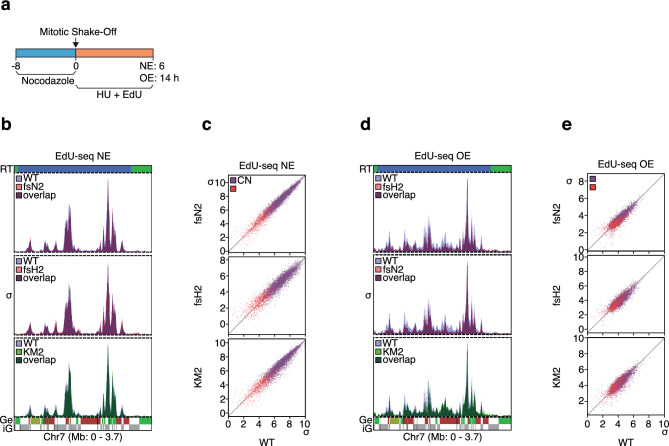
| Targeting *RECQL4* has no effect on DNA replication origin firing profiles. (**a**) Experimental setup for EdU-seq. (**b**) and (**d**) Overlay of DNA replication origin firing profiles of parental cells (WT) and *RECQL4*-mutant clones for a representative genomic region under conditions of normal Cyclin E expression (NE) (**b**) or Cyclin E overexpression (OE) (**d**). The EdU-seq data are presented as sigma (σ) values. RT; replication timing (blue, early; green, mid S phase); Ge, genes (green, forward direction of transcription; red, reverse; yellow, unspecified; blue, multiple genes within bin); iG, intergenic regions (gray). Bin resolution: 10 kb. (**c**) and (**e**) Correlation plots of the sigma values of all constitutive (CN, purple) and oncogene-induced (Oi, red) origins of the parental cells versus the *RECQL4*-mutant clones under conditions of normal Cyclin E expression (**c**) or Cyclin E overexpression (**e**).

The results presented so far suggest that RECQL4 may not affect DNA replication origin firing, although the possibility that firing of a subset of origins might be affected could not be excluded. We subjected two KO and one HD clones to EdU-seq (Fig. 2a), a high throughput sequencing method that allows us to identify the origins that fire, when cells enter S phase^42^. With this method we previously identified about 6,000 origins that map to the early S phase replicating genomic domains^38^. Of these 6,000 origins, we had classified about two thirds as constitutive (CN), as they fired with equal efficiency in cells expressing normal or high levels of Cyclin E; one sixth as oncogene-induced (Oi), as they fired with at least four-fold higher efficiency in response to Cyclin E overexpression; while the remaining one sixth fired at least two-fold more efficiently after Cyclin E overexpression, but did not reach the four-fold threshold required to be considered Oi. As in the original study, we examined here origin firing in the parental and *RECQL4* mutant clones under conditions of both normal Cyclin E expression (NE) and Cyclin E overexpression (OE).

Inspection of a representative genomic region, corresponding to the first 3.7 Mb of chromosome 7, revealed very similar origin firing profiles in the parental and *RECQL4* mutant cells, under NE conditions (Fig. 2b). A genome-wide analysis of the EdU-seq sigma values (normalized number of sequencing reads per bin divided by its standard deviation) of the peaks corresponding to all CN and Oi origins confirmed these findings (Fig. 2c). Furthermore, no differences in origin firing efficiency between the parental and *RECQL4* mutant cells were observed when cyclin E was overexpressed (Fig. 2d, e). Thus, despite the presence of an Sld2-like domain in the N-terminus of RECQL4 (Supplementary Fig. 2d) and, in contrast, to previous studies suggesting that RECQL4 is critical for origin firing in vertebrates^28–30^, the human U2OS *RECQL4* KO and HD cells did not have obvious defects in origin firing in early S phase.

### RECQL4 helicase activity affects fork progression

**Figure 3:**
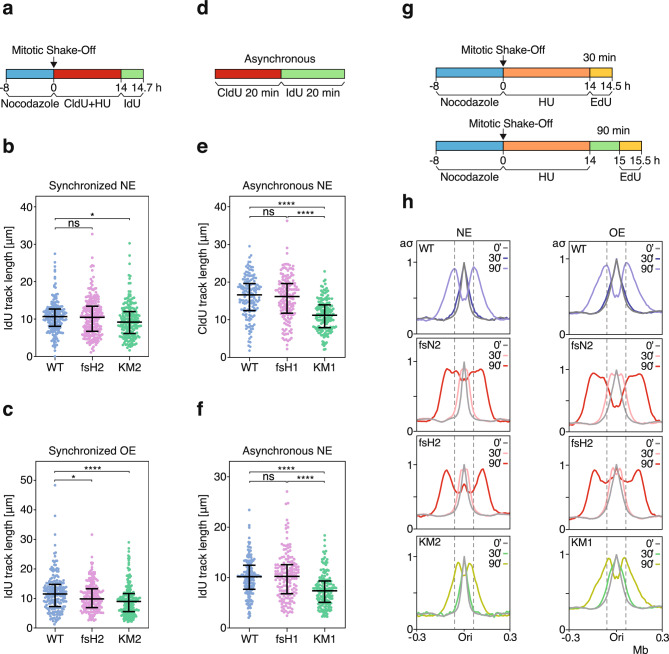
| RECQL4 affects DNA replication fork progression. (**a**) Experimental outline for the analysis of fork progression by DNA combing in synchronized cells. (**b**) and (**c**) Lengths of IdU tracks of parental cells (WT) and *RECQL4*-mutant clones treated as shown in (**a**). NE, normal levels of Cyclin E (**b**); OE, Cyclin E overexpression (**c**). More than 190 IdU tracks were measured per sample. (**d**) Experimental outline for the analysis of fork progression by DNA combing in asynchronous cells. (**e**) and (**f**) Lengths of CldU (**e**) and IdU (**f**) tracks of parental cells (WT) and *RECQL4*-mutant clones treated according to the outline shown in (**d**). The cells expressed normal levels of Cyclin E (NE). More than 150 CldU-IdU double-labeled fibers were measured per sample. For all samples, the median and upper and lower quartiles are indicated by horizontal lines. *P* values were calculated by a two-way ANOVA with Fisher’s Least Significance Difference test. *, *P* < 0.05; ****, *P* < 0.0001; ns: not significant. (**g**) Experimental outline for the study of fork progression by EdU-seq. For the 0 min (’ or min) timepoint, after mitotic shake-off, the cells were incubated with HU and EdU for 14 h. (**h**) Genome-wide averages of the EdU-seq sigma values of the indicated samples and timepoints over genomic regions spanning 0.6 Mb around origins of replication. NE, normal levels of Cyclin E; OE, overexpression of Cyclin E; aσ, adjusted sigma values relative to the no-release (0 min) samples.

Not being able to observe an effect of *RECQL4* inactivation on origin firing, we examined whether fork progression was affected. For this purpose, we first relied on single molecule DNA fiber analysis. Parental and *RECQL4* mutant cells were synchronized by mitotic shake-off and released into the cell cycle in the presence of hydroxyurea (HU) and the thymidine analog 5-chloro-2’-deoxyuridine (CldU). Fourteen hours later, when the cells had entered S phase, HU was removed and CldU was replaced by 5-iodo-2’-deoxyuridine (IdU); the cells were collected 40 min later and the lengths of the IdU-labeled DNA tracks were used to calculate fork progression rates (Fig. 3a and Supplementary Fig. 4a). The experiment was performed under conditions of normal cyclin E expression (NE) and cyclin E overexpression (OE). Under NE conditions, fork progression rates were similar in the *RECQL4* KO clone and the parental cells, but lower in the HD clone (Fig. 3b). Under OE conditions, both the KO and HD clones exhibited lower fork progression rates in comparison to the parental cells, although the decrease in the rate of fork progression was greater in the HD cells (Fig. 3c).

To rule out the possibility that the cell synchronization protocol or HU might have affected the results, we repeated the experiment with asynchronous cells, treating them with CldU and then with IdU, in the absence of HU or any other agents (Fig. 3d). In this experiment the cells expressed normal levels of Cyclin E. Similarly to the results with the synchronized cells, the KO clone exhibited similar fork progression rates as the parental cells, while the fork progression rate was reduced in the HD clone (Fig. 3e, f). The lengths of the IdU and CldU tracks were similarly affected in all these cells (Supplementary Fig. 4b).

The above experiments interrogated two *RECQL4* KO clones, fsH1 and fsH2, that showed no or little decrease in fork progression rates and two HD clones, KM1 and KM2, that showed a significant decrease in fork progression rates under both NE and OE conditions. To confirm the decrease in fork progression rates in the HD clones with a second method, we employed a variation of the EdU-seq protocol, in which synchronized cells entered S phase in the presence of HU to block fork progression and then were allowed to resume fork progression by removing HU from the media. The cells were harvested 30 and 90 after removal of the HU and the nascent DNA was labeled by adding EdU 30 min before collecting the cells (Fig. 3g). Both HD clones showed significantly decreased fork progression, irrespective of whether cyclin E was overexpressed or not (Fig. 3h and Supplementary Fig. 4c, d).

Surprisingly, the KO clones appeared to show increased rates of fork progression, under both normal and oncogene-induced replication stress conditions (Fig. 3h and Supplementary Fig. 4c, d). This apparent increase in fork progression rates was evident even 30 min after release from the HU-induced block (Fig. 3h). The easiest way to reconcile these results with the very small change in fork progression rates measured by the DNA combing assay (Fig. 3a–f), is by considering that in the KO clones the forks recover faster after HU removal than in the parental cells. HU leads to a decrease in the levels of deoxynucleotide triphosphates (dNTPs) and dNTP levels are limiting for fork progression^43–45^. If fewer origins had fired in the KO clones, as compared to the parental cells, then the forks in the KO cells would be able to resume DNA synthesis earlier after HU removal. Thus, in this case, the EdU-seq experiment may be indicating changes in fork recovery after HU removal, rather than changes in fork progression rates.

The effects of targeting *RECQL4* on fork recovery/progression prompted us to examine the length of S-phase in the KO and HD *RECQL4* mutant clones. Cells expressing normal levels of Cyclin E were released from a thymidine block and DNA content and EdU incorporation were examined 6, 9 and 10 h later (Supplementary Fig. 5a). The increase in DNA content over time, indicated that progression through S-phase was similar in the parental cells and the *RECQL4* mutant clones (Supplementary Fig. 5b, c). We also examined whether inactivation of *RECQL4* led to induction of a DNA damage response, as ascertained by expression of phosphorylated histone H2AX (γH2AX), a marker of DNA damage^46,47^. In the absence of Cyclin E overexpression, both the KO and HD clones had low levels of γH2AX, similar to the parental cells (Supplementary Fig. 6). Thus, even though targeting the *RECQL4* locus affected fork recovery/progression, it did not lead to major changes in cell cycle progression or induction of a DNA damage response.

### RECQL4 is not critical for mitotic DNA synthesis

Mitotic DNA synthesis (MiDAS) is a special kind of DNA replication that may occur when cells enter mitosis^48^. Our current understanding is that MiDAS completes replication of genomic regions that had not been replicated during interphase. Since MiDAS is a form of DNA replication, we wondered if RECQL4 might play a role in initiating MiDAS. Previous studies have demonstrated that MiDAS is induced by treating cells with the DNA polymerase inhibitor aphidicolin. Such treatment results in origin-poor genomic regions remaining unreplicated by the time cells enter mitosis and requiring MiDAS for completion of DNA replication. The genomic regions where MiDAS occurs have been identified^49,50^ and include the so-called common fragile sites (CFS), which are sites of recurrent chromosomal breaks in aphidicolin-treated cells^51^.

**Figure 4:**
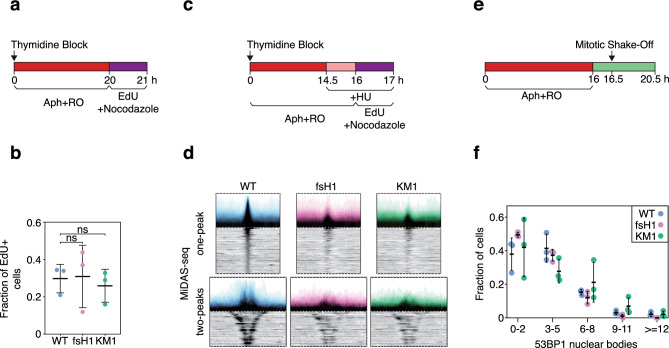
| RECQL4 is not critical for MiDAS. (**a**) Experimental outline for the assessment of the presence of MiDAS in mitotic parental (WT) and *RECQL4* mutant cells by epifluorescence microscopy. (**b**) Fraction of EdU + mitotic cells, according to (**a**); at least two EdU foci per mitosis were required to consider a cell positive for MiDAS. Means and standard deviation of three independent experiments are shown on the graph. At least 130 mitotic cells were analyzed for each condition. Statistical analysis was performed with 2-way ANOVA with Tukey’s Multiple Comparisons test. ns, not significant. (**c**) Experimental outline for the study of mitotic DNA synthesis by MiDAS-seq. (**d**) Average MiDAS-seq signal (σ values) of all MiDAS regions and heatmap of the MiDAS-seq signal of each MiDAS region ranked according to its genomic size for single- or double-peak regions. Bin resolution: 10 kb. (**e**) Experimental outline for monitoring 53BP1 nuclear bodies in G1. (**f**) Fraction of cells containing 53BP1 nuclear bodies in early G1, according to (**e**). The graph shows averages and standard deviations from three independent experiments, with at least 230 cells analyzed per cell line. No significant differences between the parental cells and *RECQL4* mutant clones were observed (2-way ANOVA with Tukey’s Multiple Comparisons test). Aph, aphidicolin; RO, RO3306.

We studied MiDAS in aphidicolin-treated parental cells and *RECQL4* mutant clones using fluorescence microscopy to monitor EdU incorporation in mitotic chromatin. No significant differences in the number of EdU-positive mitotic cells was observed between the *RECQL4* mutant and parental cells (Fig. 4a, b). These results were validated by sequencing the DNA being synthesized in mitotic cells. The genome-wide MiDAS-seq profiles were very similar in the parental and *RECQL4* mutant cells (Fig. 4c, d).

Failure to complete replication of the genome in mitosis leads to formation of 53BP1 nuclear bodies in the subsequent G1 phase of the cell cycle^52,53^. We therefore also monitored the presence of 53BP1 bodies in *RECQL4* mutant and parental aphidicolin-treated cells. In further support of the conclusion that RECQL4 is not required for MiDAS, we observed no difference in 53BP1 bodies in the parental and *RECQL4* mutant cells (Fig. 4e, f).

## Discussion

Eukaryotic DNA replication is a highly regulated process, whose fidelity is critical for preserving the integrity of the genome. A key aspect of the regulation of eukaryotic replication is that origins are licensed in G1, but fire in S phase. The temporal separation of origin licensing and firing ensures that origins fire only once per cell cycle, which is important to prevent the emergence of focal genomic duplications. Origin firing is induced by S phase-specific cyclin-dependent kinases (S-CDKs)^11^. In budding yeast, S-CDKs phosphorylate Sld2 and Sld3, leading to formation of a ternary Sld2-Dpb11-Sld3 complex that is required for origin firing^9,10^. By analogy to yeast, it has been proposed that in higher eukaryotes RECQL4, TRESLIN and TOPBP1, the presumed orthologs of Sld2, Sld3 and Dpb11, respectively, would form a ternary complex that stimulates origin firing. Indeed, TRESLIN is phosphorylated by CDKs and its phosphorylation facilitates its interaction with TOPBP1 and origin firing^18,54,55^. However, the residue of budding yeast Sld2 that is phosphorylated by S-CDKs, is not conserved in human RECQL4^11^.

In this study we show that human RECQL4 is not critical for origin firing, contrary to the function of Sld2 in budding yeast. Cells that did not express detectable levels of RECQL4 were viable, had normal kinetics of entry into S-phase and their DNA replication origin firing profiles were indistinguishable from those of wild-type cells. In agreement with our findings, large-scale high-throughput RNAi and CRISPR screens, whose results are included in the DEPMAP RNAi databases^56,57^ and the CRISPR screens section of the BioGRID database^58^, reveal that, in the majority of cell lines, cell proliferation is not significantly impacted upon disruption of *RECQL4* expression, whereas it is impacted upon disruption of *TOPBP1* expression. Nevertheless, there are also several publications suggesting that RECQL4 is important for initiation of DNA replication in *C. elegans*, *Drosophila*, *Xenopus*, chicken, mouse and human cells^20,21,24–30^. In some of these studies, *RECQL4* was shown to have a strong effect on initiation of DNA replication, while in other studies the effect was milder. For example, in one study in *Drosophila*, knock-out of the *RECQL4* gene led to embryonic lethality, but the null homozygote embryos survived to the larval stage^25^, implying many rounds of DNA replication in the apparent absence of RECQL4. The best way to reconcile our findings with the previous reports in metazoans is to consider that RECQL4 has a role in initiation of DNA replication, but that this role in some settings is not critical, such that cells, in which the *RECQL4* locus is targeted, can proliferate normally. Another possibility is that RECQL4 is required for firing of origins in mid or late S phase, but not in early S phase. Since the EdU-seq method identifies only the origins that fire in early S phase, our study would have missed such a function. Finally, we note that it is possible that very low levels of truncated, yet functional, RECQL4 polypeptides might still be expressed in the *RECQL4* KO clones described here. If this were the case, then very low levels, almost undetectable levels, of truncated RECQL4 would suffice for origin firing. In the fsN clones, the entire Sld2-like domain of RECQL4 is not deleted, but the most highly conserved residues between RECQL4 and Sld2 are targeted by the CRISPR-induced mutagenesis.

Our findings are consistent with some aspects of origin firing having diverged in evolution, such that metazoan RECQL4 has a somewhat different role than budding yeast Sld2, despite their sequence similarity. Indeed, budding yeast Sld2 forms a pre-LC together with GINS, Dpb11 and DNA polymerase epsilon and is important for recruiting GINS to the MCM^7,8^, whereas, *Xenopus* RECQL4 acts after GINS has been recruited to the MCM^20,21^. The function of Sld2 appears more similar to that of DONSON, a metazoan protein that forms a complex with GINS and TOPBP1 and recruits GINS to the MCM^31–36^. As a result of this functional divergence in origin firing, RECQL4 may have become less critical for initiation of DNA replication than budding yeast Sld2.

## Methods

### Cell culture

Cyclin E-inducible (tet-off system) U2OS (U2OS CycE) cells, kindly provided by Prof. J. Bartek, were cultured in Dulbecco’s modified Eagle’s medium (Invitrogen, Cat. No. 11960), supplemented with 10% fetal bovine serum (FBS; Invitrogen, Cat. No. 10500), penicillin/streptomycin/glutamine (Invitrogen, Cat. No. 10378–016) with 1 µg/ml puromycin (Sigma, Cat. No. P8833) and 400 µg/ml G418 (Invitrogen, Cat. No. 10131–027) in presence or absence of 2 µg/ml tetracycline (Sigma, Cat. No. T7660) to suppress or induce, respectively, expression of an ectopic *CCNE1* gene, as previously described^37^.

### Oligonucleotide sequences

Guide RNA *RECQL4* exon 1: 5’-CGTGGGAGCGCGCGTTCCGA-3’.

Guide RNA *RECQL4* exon 9: 5’-CACGTTGGTCGTCTCTCCCC-3’.

Guide RNA *RECQL4* exon 9 K508M: 5’-CTGGTAGCACAGGGACTTGC-3’.

ssDNA repair template K508M (lowercase indicate mutated nucleotides):

5’-CCCATCCAGGCATCTCCACGCTGCTGGTGCTGCCTACAGGaGCtGGgATGTCCC TGTGCTACCAGCTCCCAGCGCTGCTCTACAGCCGGC-3’.

Primer forward K508M screen WT: 5’-TGCTGCCTACAGGTGCCGGCAA-3’.

Primer forward K508M screen mutation: 5’-TGCTGCCTACAGGaGCtGGgAT-3’; lowercase indicates mutated nucleotides.

Primer reverse K508M screen WT: 5’-GAGTCACAAGTGCTGGTTCTTG-3’.

### Engineering *RECQL4* KO and HD clones

*RECQL4* KO U2OS CycE clones were generated by transfecting U2OS CycE cells with a pX458_CRISPR_GFP vector targeting either the first (guide RNA *RECQL4* exon 1) or the ninth exon (guide RNA *RECQL4* exon 9) of the *RECQL4* gene using an Amaxa® Cell Line Nucleofector® Kit V (Lonza) according to the manufacturer’s instructions (3 µg vector for 10^6^ cells). Three days later, GFP-positive cells were sorted to allow the expansion of single-cell clones. KO clones were selected by western blotting for RECQL4 and validated by Sanger sequencing of the targeted *RECQL4* locus.

*RECQL4* HD U2OS CycE clones substituted lysine 508 of RECQL4 with a methionine. Cells were co-transfected with 0.8 µg pX458_CRISPR_GFP vector targeting the ninth exon of *RECQL4* near the codon of lysine 508 (guide RNA *RECQL4* exon 9 K508M) and 1.3 µl ssDNA 100 µM repair template (DNA repair template K508M); three days later, GFP-positive cells were sorted for clonal expansion. Single-cell clones were screened by PCR to detect the K508M mutation using the AccuPrime™ Pfx SuperMix kit (Invitrogen) according to the manufacturer’s instructions. Selected clones were validated by Sanger sequencing.

### siRNA transfection

Cells were transfected with siRNA targeting *RECQL4* (Dharmacon, Cat. No. L-010559–00) and *TICRR* (GACCUGAGAGAAGAUUCAGAAGUUA, Kumagai et al., 2010^54^) using the INTERFERin transfection reagent (Polyplus, Cat. No. 101000016) according to the manufacturer’s instructions. Three days later, the cells were subjected to an EdU (Invitrogen Cat. No. A10044) pulse (10 µM) for 30 min before collection. They were subsequently fixed with 90% methanol and stored at − 20 °C overnight for flow cytometry analysis.

### Plasmid electroporation

Cells were transfected with plasmids containing the coding sequence of the human *RECQL4* gene corresponding to the first 321 or 371 amino acids of RecQL4 (pCDZ2FE_RecQL4_1-321 and pCDZ2FE_RecQL4_1-371; human *RECQL4* gene fragment cloned in pcDNA3.1-zeocin vector containing 2 N-terminal FLAG tags and a small Gly-rich linker) respectively, or with a plasmid containing the full-length coding sequence of *RECQL4* (p3XFLAG-mycCMV23_RecQL4; human *RECQL4* gene cloned in p3XFLAG-myc-CMV-23 vector containing an N-terminal 3xFLAG tag and a C-terminal myc tag). The electroporation was performed with the Amaxa Cell Line Nucleofector Kit V (Lonza, Cat. No. VCA-1003) using an Amaxa Nucleofector II electroporation apparatus, according to the manufacturer's instructions.

### Flow cytometry

Flow cytometry was performed as previously described^38^. Briefly, cells were stained for EdU (Invitrogen, Cat. No. A10044) incorporation by linking an Alexa Fluor 647 Azide (Thermo Fisher Scientific, Cat. No. A10277) to the incorporated EdU using Click-it chemistry (Invitrogen, Cat. No. C-10420). Genomic DNA was stained with propidium iodide (Sigma, Cat. No. 81845) after treatment of the cells with DNase-free RNase (Roche, Cat. No. 11119915001). Data were acquired on a Gallios flow cytometer (Beckman Coulter) and analyzed by Kaluza flow cytometry software (Beckman Coulter).

### Antibodies, immunoblotting and immunofluorescence

Immunoblotting was performed as previously described^59^. Antibodies specific for RECQL4 (SDIX, Cat. No. 2547.00.02; Novus Biologicals, Cat. No. NBP2-47,310), Cyclin E (DBS, Cat. No. Mob181), PCNA (Genetex, Cat. No. GTX-20029), Treslin (Abcam, Cat. No. ab124268) and Lamin A (Abcam, Cat. No. ab26300) were used at the concentration recommended by the manufacturer.

To detect 53BP1 bodies by immunofluorescence, cells grown on coverslips were fixed for 30 min in 4% paraformaldehyde and then permeabilized with 0.2% Triton X-100 in PBS for another 30 min. Blocking was performed with 1% BSA in PBS for 1 h at room temperature followed by incubation for 1 h at room temperature with an in-house mouse monoclonal antibody raised against human 53BP1^60^ and, then, for 30 min at room temperature, with a secondary, fluorophore-conjugated antibody (Invitrogen). Finally, the cells were counterstained with DAPI (1 μg/ml in water) for 1 min. The coveslips were mounted on glass slides with Fluoromount-G™ (Invitrogen, Cat. No. 00-4958-02) mounting medium and stored overnight for the medium to seal. Images were acquired on a Zeiss, AxioImager M.2 microscope, equipped with an Apotome module. Image analysis was performed with Fiji^61^ or the proprietary Zen software (Carl Zeiss). Data analysis and plotting was performed with GraphPad Prism.

### Cell cycle kinetics

To monitor the kinetics of entry into S phase, asynchronous cells were exposed to nocodazole (0.1 μg/ml; Tocris, Cat. No. 1228) for 8 h, mitotic cells were obtained by mitotic shake-off, nocodazole was washed away and the cells were incubated in media containing EdU (10 µM) for the indicated periods of time. The cells were harvested and DNA content and EdU incorporation were quantified by flow cytometry, as described above.

To study progression through S-phase, cells were synchronized at the G1/S boundary by a thymidine block (2 mM thymidine for 18 h) and then released into S-phase by washing the thymidine away (3 washes with warm 1X PBS). The cells were harvested 6, 9 or 10 h later; EdU was added to the media 30 min before harvesting. DNA content and EdU incorporation were quantified by flow cytometry, as described above.

### EdU-seq

EdU-seq experiments were performed as previously described^38,42^. For the experiments monitoring firing of DNA replication origins, cells collected by mitotic shake-off were released into the cell cycle in media containing EdU (Invitrogen, Cat. No. A10044) and HU (Sigma, Cat. No. H8627). The cells were harvested at the indicated times and permeabilized; the incorporated Edu was conjugated to a cleavable biotin-azide linker (azide-PEG(3 + 3)-S–S-biotin; Jena Biosciences, Cat. No. CLK-A2112-10) by a Click-It reaction. Genomic DNA was extracted, sonicated and EdU-positive DNA fragments were captured using Dynabeads MyOne Streptavidin C1 (Invitrogen, Cat. No. 65001). Next generation library preparation and sequencing were performed at the iGE3 genomics platform of the University of Geneva.

For the experiments to monitor fork progression by EdU-seq, cells, synchronized by mitotic shake-off, were incubated with HU for the indicated periods of time; HU was washed away and the cells were allowed to proceed into S-phase for the indicated periods; EdU was added to the media 30 min before harvesting the cells. All the subsequent steps were as described above.

### MiDAS-seq

MiDAS-seq was performed as described previously^49^. Briefly, asynchronous cells were subjected to a single thymidine (2 mM for 18 h) block (Sigma-Aldrich, Cat. No. T1895) and then released into S-phase by washing the thymidine away with three warm 1X PBS washes; after release from the thymidine block, the cells were incubated in media supplemented with 0.4 μM aphidicolin (Sigma-Aldrich, Cat. No. A0781) and 9 μM RO3306 (APExBio, Cat. No. A8885) for 14.5 h; 2 mM HU was then added to the media for a further 1.5 h incubation. The cells were then washed three times with warm DMEM, and allowed to enter mitosis in the presence of 200 ng/mL nocodazole, 10 μM EdU and 2 mM HU. Mitotic cells were collected one hour later by mitotic shake-off and fixed in 90% methanol at − 20 °C for at least 4 h; the cells were then treated, as described above, for the EdU-seq protocol.

### MiDAS monitoring by immunofluorescence

An immunofluorescence-based protocol to assess MiDAS was performed as previously described^48^ with minor modifications. Briefly, cells were treated with 0.4 μM aphidicolin and 9 μM RO3306 for 20 h. They were then washed three times with warm DMEM and released in mitosis in the presence of 200 ng/mL nocodazole and 25 μM EdU for one hour. The cells were then fixed for 20 min in PTEMF buffer (20 mM PIPES pH 6.8, 10 mM EGTA, 0.2% Triton X-100, 1 mM MgCl_2_ and 4% formaldehyde) and further permeabilized with 0.2% Triton X-100 for another 30 min at room temperature. Samples were subjected to Click-It reaction with Alexa Fluor 647 Azide (Thermo Fisher Scientific, Cat. No. A10277) and counterstained with DAPI. Images were acquired using an epifluorescence microscope (Zeiss, AxioImager M.2, equipped with an Apotome module) with an oil immersion × 100 objective using the ‘Z-stack’ function with a 0.5 μm step. The orthogonal projections were scored for EdU foci on prometaphase chromatin.

### γH2AX staining

Asynchronous cells were harvested by trypsinization and fixed in 70% ethanol overnight. They were, subsequently, stained for γH2AX with the Luminex Guava Histone H2A.X Phosphorylation Assay Kit (Cat. No. FCCS100182) according to the manufacturer’s directions. The samples were analyzed by flow cytometry, as described above.

### 53BP1 bodies

Asynchronous cells were treated with 0.4 μM aphidicolin and 9 μM RO3306 for 16 h and then washed three times with warm tissue-culture medium; 30 min later, the mitotic cells were collected by mitotic shake-off and re-seeded on plates in fresh medium. 53BP1 bodies were scored 4 h later by immunofluorescence, as described above.

### DNA single molecule fiber analysis

Cells were incubated for the indicated time periods with 40 μΜ CldU (Sigma-Aldrich, Cat. No. C6891), washed three times with warm PBS and then incubated with 400 μΜ IdU (Sigma-Aldrich, Cat. No. I7125). After the end of the second incubation period, the cells were collected by trypsinization and cell pellets were stored at − 20 °C. Combing was performed with the FiberPrep® DNA extraction Kit (Genomics Vision, Cat. No. EXT-001) following the manufacturer’s instructions. Briefly, 2*10^5^ cells per sample were placed in agarose plugs and subjected to Proteinase K digestion overnight. The following day, the plugs were washed three times with a proprietary Wash buffer on a rotating wheel for one hour per wash; the plugs were then transferred to 2 ml, round bottom, low DNA binding tubes (Eppendorf, Cat. No. 022431048) and allowed to melt at 68 °C for 20 min. The plugs were subsequently equilibrated at 42 °C for 10 min, β-agarase was added and the samples were incubated overnight at 42 °C. The following day, DNA fibers were combed onto silanized coverslips (Genomics Vision, Cat. No. COV-002-RUO) with a custom-made machine that allows the retraction of the coverslip from the sample at a constant rate of 300 μm/s. The combed coverslips were heated at 60 °C for 2–4 h and stored at − 20 °C.

Immunodetection of the labelled DNA was performed as previously described^62^. Briefly, the coverslips were mounted facing up onto glass slides using commercial super-glue. They were subjected to baths of increasing concentration of Ethanol (70, 90, 100%) in Coplin jars, for 5 min each, to dehydrate. After being completely air dried, the DNA was denatured with 1 M NaOH, for exactly 25 min. Following the denaturation, the slides were neutralized with PBS (5 washes, 1 min each) and blocked with 1% BSA, 0.1% Triton X-100 in PBS for 15 min at room temperature. For immunodetection of the thymidine analogs, the following antibodies were used: for CldU, rat monoclonal anti-BrdU diluted 1:200 (Abcam, Cat. No. BU1/75-ICR1); for IdU, mouse monoclonal anti-BrdU, diluted 1:50 (Beckton Dickinson, Cat. No. 347580); for the DNA fibers, anti-ssDNA, diluted 1:20 (IBL, Cat. No. 18731). All primary antibodies were diluted in PBS supplemented with 0.1% Triton X-100 (PBS/T) and were incubated with the samples overnight at 4 °C. The following day the samples were washed twice with PBS/T for 5 min per wash and incubated with the recommended secondary antibodies at room temperature for one hour, followed by 3 quick washes with PBS/T and air drying. Glass coverslips were mounted on top of the combed coverslips with Fluoromount-G™ (Invitrogen, Cat. No. 00-4958-02) and allowed to harden overnight before images were acquired by a Hamamatsu camera attached to an epifluorescence microscope (Zeiss, AxioImager M.1 or M.2, equipped with a motorized table) with an oil immersive × 100 objective. Image analysis was performed as described above.

### Analysis of high throughput sequencing data

The sequencing data were processed as described previously^38,42^. Briefly, the sequencing reads were aligned on the human genome assembly (GRCh37/hg19) using the Burrows–Wheeler aligner algorithm and retaining only the reads with the highest quality score. The genome was divided in 10 kb bins and previously described custom Perl scripts were used to calculate sigma values for each genomic bin. The sigma value is a measure of peak height and indicates the normalized number of sequencing reads per bin divided by its standard deviation. The normalization of the sequence reads was performed by dividing the number of obtained sequence reads in each genomic bin by the number of sequencing reads at that genomic bin obtained previously by deep sequencing of genomic DNA prepared from the same cell line. This normalization adjusts for biases in library preparation and for differences in the fraction of masked sequences at each genomic bin. Custom Perl scripts were used to visualize the data^38,42^. BigWig files were generated with deepTools v3.5.1.

For genome-wide comparisons between the parental cells and the *RECQL4* mutant clones, we utilized the list of DNA replication origins determined previously in the same parental cells expressing either normal levels of Cyclin E (NE) or after inducing overexpression of Cyclin E (OE). These origins were annotated as constitutive (CN), oncogene-induced (Oi) and intermediate (also referred to as Oi2) depending on their sigma values in the cells with and without Cyclin E overexpression^38^. For the genome-wide comparisons, the sigma values of all DNA replication origins of the parental cells were plotted against the corresponding values of each *RECQL4* mutant clone.

For visualization of the MiDAS-seq data, we focused on 293 genomic regions previously identified as sites of MiDAS in aphidicolin-treated U2OS cells^49^. These genomic regions were previously classified as double-peak or single-peak, depending on the number of peaks present in each genomic region, which in turn depends on the length of the genomic region that has not been replicated, when cells enter mitosis. For each dataset, we plotted the average MiDAS-seq signal of all MiDAS regions and below that a heatmap of the MiDAS-seq signal of each MiDAS region ranked according to its genomic size for double-peak regions or its sigma value for single-peak regions.

### Supplementary Information

Supplementary Information.

## Data Availability

The EdU-seq and MiDAS-seq data reported in this article have been submitted to the GEO database with accession numbers: GSE225532.
