# Supplementary material for: RECQL4 is not critical for firing of human DNA replication origins
Source: Sci Rep. 2024 Apr 2;14:7708. doi: 10.1038/s41598-024-58404-0 (PMC10987555; doi:10.1038/s41598-024-58404-0)
Supplement: Supplementary file 1 — Supplementary Information. [file 41598_2024_58404_MOESM1_ESM.pdf]

Supplementary Information

RECQL4 is not critical for firing of human DNA replication origins

Laura Padayachy[1&], Sotirios G. Ntallis[1&] and Thanos D. Halazonetis[1*]

[1]Department of Molecular and Cellular Biology, University of Geneva, 1205 Geneva, Switzerland.

[&]Equal contribution.

[*]email: thanos.halazonetis@unige.ch

Supplementary Figure 1

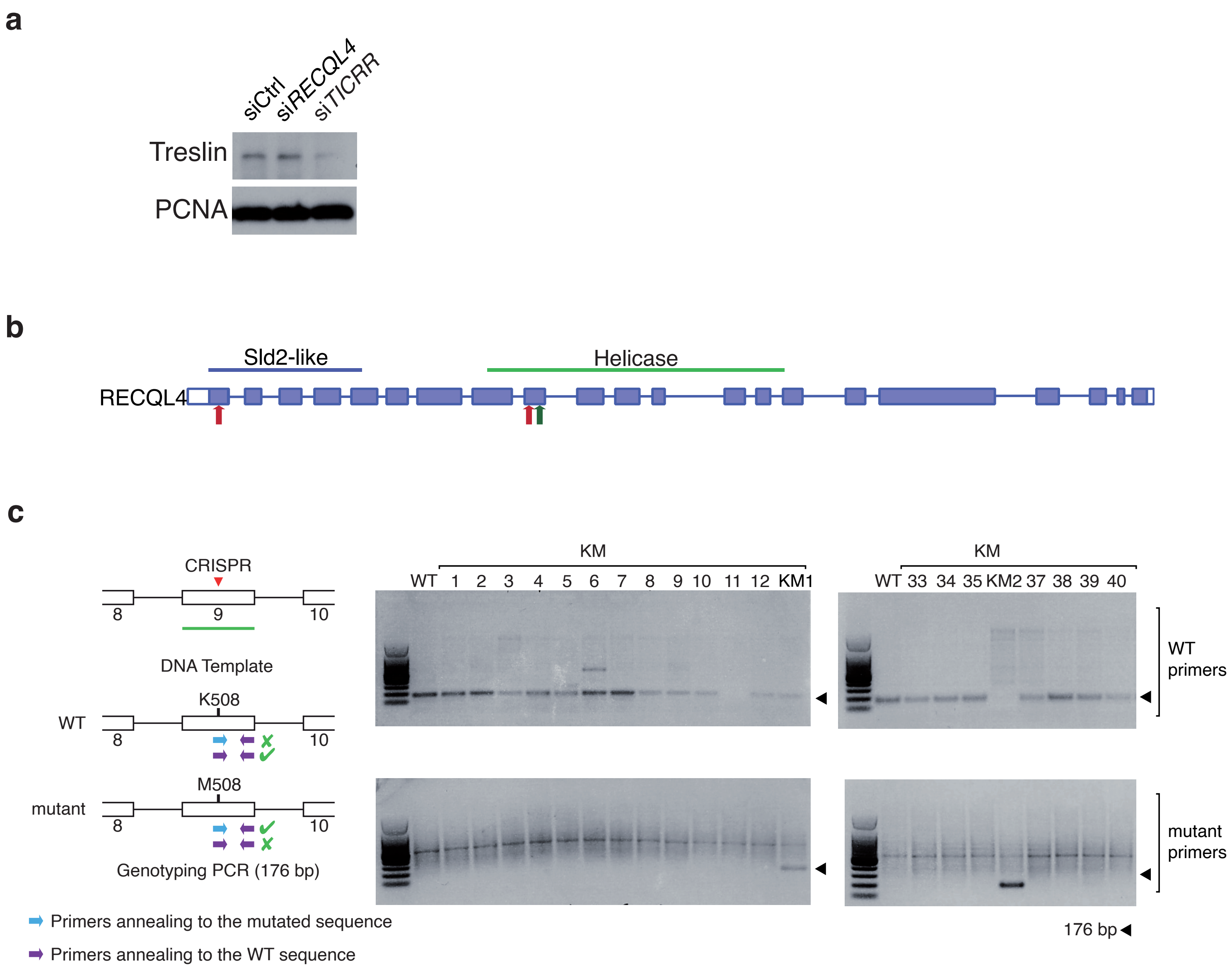

Supplementary Fig. 1 | Validation of TRESLIN depletion by siRNA and generation and screening of *RECQL4* clones.

a Immunoblot showing depletion of TRESLIN by specific siRNA; PCNA serves as a loading control. **b** Graphical representation of the exons of the *RECQL4* gene. Coding regions are drawn as filled boxes; non-coding exonic regions as open boxes. Red and green arrows indicate the positions targeted by the CRISPR guide RNAs used to generate the KO and HD clones, respectively. **c** PCR-based genotyping to screen the HD clones. Left, genotyping strategy; Right, analysis of a panel of putative HD clones. On the top panels, a PCR primer set recognizing the WT sequences with an expected amplicon size of 176bp was used. On the bottom panels, the forward primer was substituted with one bearing the expected mutations for the HD clones. The arrows indicate the expected amplicon size from each PCR reaction.

Supplementary Figure 2

a

fsN1

```
                        1 M  E  R  L  R  D  V  R  E  R  L  Q  A  W  E  R  A  F  R  R  Q  R  G  R  R  P  S  Q  Intron---------- 28
WT              145743168 ATGGAGCGGCTGCGGGACGTGCGGGAGCGGCTGCAGGCGTGGGAGCGCGCGTTCCGACGGCAGCGCGGGCGGCGACCGAGCCAGGTGCGGGCTGCCCAGG 145743071
                          ||||||||||||||||||||||||||||||||||||||||||||||||                 |||||||||||||||||||||||||||||||||
  clone allele 1          ATGGAGCGGCTGCGGGACGTGCGGGAGCGGCTGCAGGCGTGGGAGCGC-----------------AGCGGCGACCGAGCCAGGTGCGGGCTGCCCAGG
                        1 M  E  R  L  R  D  V  R  E  R  L  Q  A  W  E  R                  A  A  T  E  P  G Intron---------- 22

                        1 M  E  R  L  R  D  V  R  E  R  L  Q  A  W  E  R  A  F  R    R  Q  R  G  R  R  P  S  Q  Intron-------- 28
WT              145743168 ATGGAGCGGCTGCGGGACGTGCGGGAGCGGCTGCAGGCGTGGGAGCGCGCGTTCC--GACGGCAGCGCGGGCGGCGACCGAGCCAGGTGCGGGCTGCCCA 145743069
                          |||||||||||||||||||||||||||||||||||||||||||||||||||||||  |||||||||||||||||||||||||||||||||||||||||||
  clone allele 2          ATGGAGCGGCTGCGGGACGTGCGGGAGCGGCTGCAGGCGTGGGAGCGCGCGTTCCCCGACGGCAGCGCGGGCGGCGACCGAGCCAGGTGCGGGCTGCCCA
                        1 M  E  R  L  R  D  V  R  E  R  L  Q  A  W  E  R  A  F  P  D  G  S  A  G  G  D  R  A  R Intron-------- 29
```

fsN2

```
                       15 E  R  A  F  R---------------------------------//----------------------------------------------R  Q 21
WT              145743126 GAGCGCGCGTTCC---------------------------------//-----------------------------------------------GACGGC 145743108
                          |||||||||||||
  clone allele 1+2        GAGCGCGCGTTCCCCTAACCCTAACCCTAACCCTAACCCTAACCCT//CTAACCCTAACCCTAACCCTAACCCTAACCCTAACCCTAACCCTACGACGGC
                       15 E  R  A  F  P  *                                                                                        19
```

b

fsH1

```
                      514 L  P  A  L  L  Y  S  R  R  S  P  C  //Intron------------------------------------------------------ 525
WT              145740400 CTCCCAGCGCTGCTCTACAGCCGGCGCAGCCCCTGC//GGCAGCCCAGCTCCACAGGCACCACAGCCACCCCTACGGGAAATGTGCTGGGAAAGGAGCCA 145740107
                          ||||||||||||||||||||||||||||||||||||||||||||||||||||||
  clone allele 1          CTCCCAGCGCT-------------------------//------------------GCACCACAGCCACCCCTACGGGAAATGTGCTGGGAAAGGAGCCA
                      514 L  P  A  L -------------------------//--------------------------------------------------------------- 517

                      514 L  P  A  L  L  Y  S  R  R  S  P  C  L  T  L  V  V  S  P  L  L  S  L  M  D  D  Q  Intron------------ 542
WT              145740400 CTCCCAGCGCTGCTCTACAGCCGGCGCAGCCCCTGCCTCACGTTGGTCGTCTCTCCCCTGCTGTCACTCATGGATGACCAGGTGTGCACACAGGGCCCTG 145740301
                          ||||||||||||||||||||                                  ||||||||||||||||||||||||||||||||||||||||||||
  clone allele 2          CTCCCAGCGCTGCTCTACAG----------------------------------CCCCTGCTGTCACTCATGGATGACCAGGTGTGCACACAGGGCCCTG
                      514 L  P  A  L  L  Y  S                                    P  C  C  H  S  W  M  T  R Intron------------ 529
```

fsH2

```
                      514 L  P  A  L  L  Y  S  R  R  S  P  C  L  T  L  V  V  S  P  L  L  S  L  M  D  D  Q                     542
WT              145740400 CTCCCAGCGCTGCTCTACAGCCGGCGCAGCCCCTGCCTCACGTTGGTCGTCTCTCCCCTGCTGTCACTCATGGATGACCAG                    145740320
                          ||||||||||||||||||||||||||||||||||||||||||  |||||||||||||||||||||||||||||||||||||
  clone allele 1          CTCCCAGCGCTGCTCTACAGCCGGCGCAGCCCCTGCCTCACG--GGTCGTCTCTCCCCTGCTGTCACTCATGGATGACCAG
                      514 L  P  A  L  L  Y  S  R  R  S  P  C  L  T     G  R  L  S  P  A  V  T  H  G  *                       537

                      514 L  P  A  L  L  Y  S  R  R  S  P  C  L  T  L  V  V  S  P  L  L  S  L  M  D  D  Q  Intron------------ 542
WT              145740400 CTCCCAGCGCTGCTCTACAGCCGGCGCAGCCCCTGCCTCA---------------------------------CGTTGGTCGTCTCTCCCCTGCTGTCAC 145740334
                          ||||||||||||||||||||||||||||||||||||||||                                  ||||||||||||||||||||||||||
  clone allele 2          CTCCCAGCGCTGCTCTACAGCCGGCGCAGCCCCTGCCTCACaccctaaccctaaccctaaccctaaccctaaccGTTGGTCGTCTCTCCCCTGCTGTCAC
                      514 L  P  A  L  L  Y  S  R  R  S  P  C  L  T  P  *                                                        528
```

c

KM1

```
                      510 L  C  Y  Q  L  P  A  L  L  Y  S  R  R  S  P  C  L  T  L  V  V  S  P  L  L  S  L  M  D  D  Q  Intron- 540
WT              145740412 CTGTGCTACCAGCTCCCAGCGCTGCTCTACAGCCGGCGCAGCCCCTGCCTCACGTTGGTCGTCTCTCCCCTGCTGTCACTCATGGATGACCAGGTGTGCA 145740313
                          |||||||||||||||||||||||||||||||||||||||| ||| |||||||||||||||||| ||||||||||||||||||||||||||||||||||||
  clone allele 1          CTGTGCTACCAGCTCCCAGCGCTGCTCTACAGCCGGCGCAG-CCCNGCCTCACGTTGGTCGTCTNTCCCCTGCTGTCACTCATGGATGACCAGGTGTGCA
                      510 L  C  Y  Q  L  P  A  L  L  Y  S  R  R  S  X  A  S  R  W  S  S  X  P  C  C  H  S  W  M  T  R Intron- 540

                      496 I  S  T  L  L  V  L  P  T  G  A  G  K  S  L  C  Y  Q  L  P  A  L  L  Y  S  R  R  S  P  C  L  T  L  V 529
WT              145740454 ATCTCCACGCTGCTGGTGCTGCCTACAGGTGCCGGCAAGTCCCTGTGCTACCAGCTCCCAGCGCTGCTCTACAGCCGGCGCAGCCCCTGCCTCACGTTGG 145740355
                          |||||||||||||||||||||||||||||| || || | ||||||||||||||||||||||||||||||||||||||||||||||||||||||||||||||
  clone allele 2          ATCTCCACGCTGCTGGTGCTGCCTACAGGAGCTGGGATGTCCCTGTGCTACCAGCTCCCAGCGCTGCTCTACAGCCGGCGCAGCCCCTGCCTCACGTTGG
                      496 I  S  T  L  L  V  L  P  T  G  A  G  M  S  L  C  Y  Q  L  P  A  L  L  Y  S  R  R  S  P  C  L  T  L  V 529
```

KM2

```
                      496 I  S  T  L  L  V  L  P  T  G  A  G  K  S  L  C  Y  Q  L  P  A  L  L  Y  S  R  R  S  P  C  L  T  L  V 529
WT              145740454 ATCTCCACGCTGCTGGTGCTGCCTACAGGTGCCGGCAAGTCCCTGTGCTACCAGCTCCCAGCGCTGCTCTACAGCCGGCGCAGCCCCTGCCTCACGTTGG 145740355
                          |||||||||||||||||||||||||||||| || || | ||||||||||||||||||||||||||||||||||||||||||||||||||||||||||||||
  clone allele 1+2        ATCTCCACGCTGCTGGTGCTGCCTACAGGAGCTGGGATGTCCCTGTGCTACCAGCTCCCAGCGCTGCTCTACAGCCGGCGCAGCCCCTGCCTCACGTTGG
                      496 I  S  T  L  L  V  L  P  T  G  A  G  M  S  L  C  Y  Q  L  P  A  L  L  Y  S  R  R  S  P  C  L  T  L  V 529
```

d

```
Human WT RECQL4      11 LQAWERAFRRQRGRRPSQDDVEAAPEETRALYREYRTLKRTTGQAGGGLRSSES--LPA-------AAEEAPEPRCWG 79
                        L+ WE  F  +  R P++DD+++     R +Y++Y TLK+         + + ES  LPA         E  P P+ +G
S. cerevisiae Sld2   13 LKTWEHDFIDKNKREPTRDDIKSL-RTVRQMYKQYSTLKKKQSLQRQKVDTQESVELPAHKKDHDEVVEIGPTPQVYG 89

Human WT RECQL4      82 LNRAATKSPQSTPGRSRQGSV 102
                        LN ++T++P S+P ++R G +
S. cerevisiae Sld2  161 LNASSTRTPTSSPCKNRNGKL 181
```

Supplementary Fig. 2 | Validation of the *RECQL4* mutant clones by Sanger sequencing.

a to **c** Alignment of the sequences of the *RECQL4* alleles of the validated KO clones targeting exon 1 (**a**) and exon 9 (**b**) and of the HD clones (**c**) to the wild-type *RECQL4* sequence. The amino acid sequences encoded by the gene sequences are shown using single-letter amino acid symbols; asterisks indicate stop codons. **d** Sequence similarity of the N-terminal domain of human RECQL4 to budding yeast Sld2.

Supplementary Figure 3

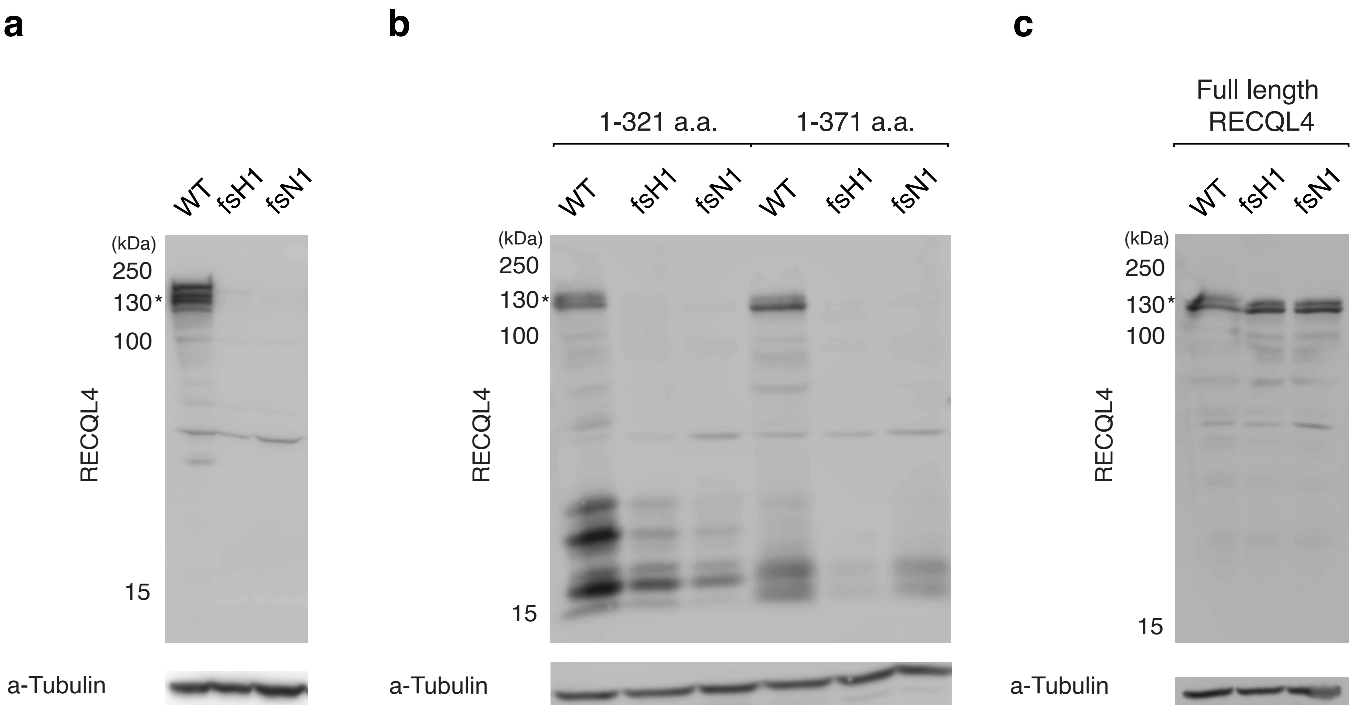

Supplementary Fig. 3 | Validation of KO clones by immunoblotting.

a Immunoblots of whole cell extracts from parental (WT) and selected *RECQL4* KO cells with an antibody that recognizes RECQL4 (clone NBP2-47310). The same samples were immunoblotted for tubulin as a control. **b** and **c** Immunoblots of whole cell extracts from WT and *RECQL4* KO cells transfected with plasmids expressing the N-terminal 321 or 371 amino acids of human RECQL4 (**b**) or full-length RECQL4 (**c**). The blots were probed with antibody clone NBP2-47310; the same samples were also immunoblotted for tubulin as a control. The positions of molecular weight markers (250, 130, 100, 15 kDa) are shown on the left of the blots. Full-length RECQL4 migrates close to the 130 kDa marker; the ectopically expressed truncated RECQL4 proteins (residues 1-321 and 1-371) migrate above the 15 kDa marker as 2-3 distinct bands indicating that they are partially proteolysed.

Supplementary Figure 4

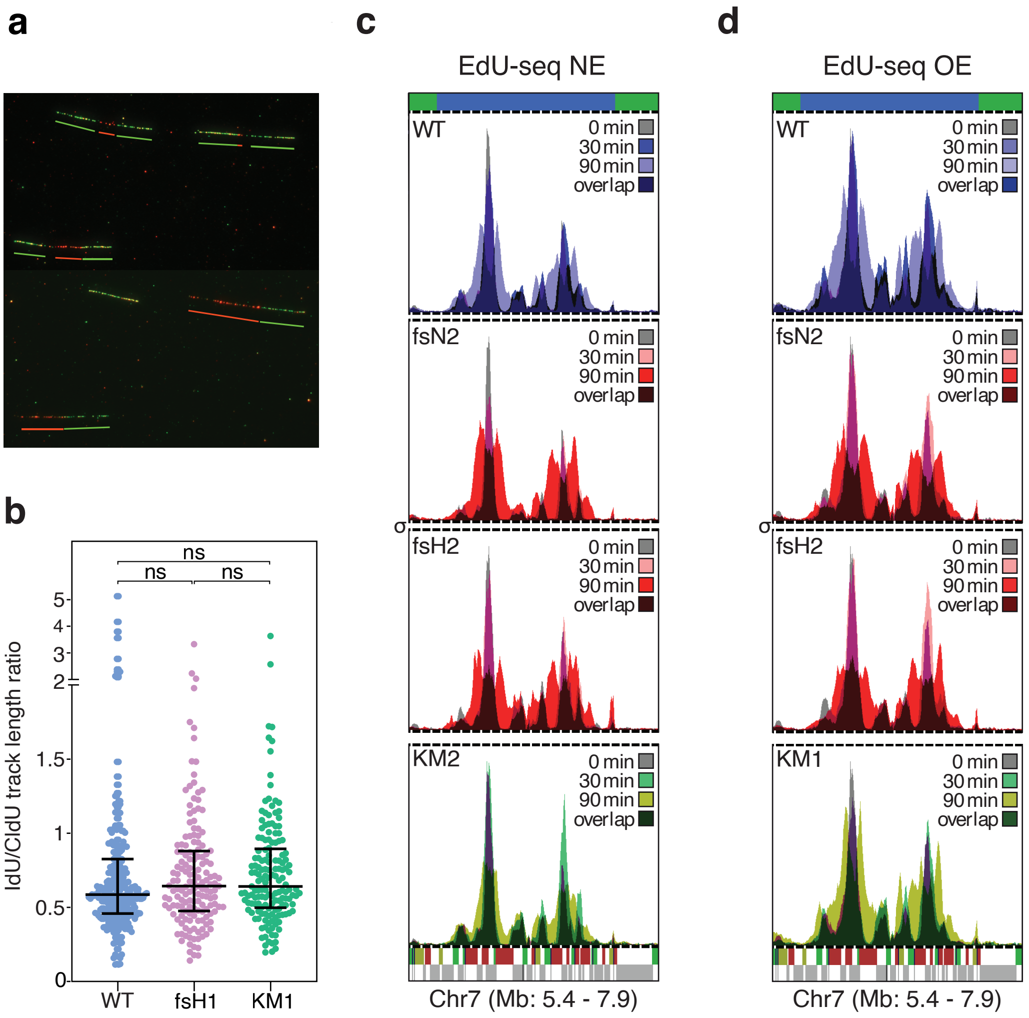

Supplementary Fig. 4 | Fork progression rates of *RECQL4* WT and mutant clones.

a Representative images of DNA combing with idealized tracks shown with solid lines below the actual images. CldU tracks are labelled red and IdU tracks green. For fork progression experiments (Fig. 3a-c), all green tracks adjacent to red tracks were evaluated. For the experiments in Fig. 3d-f where IdU to CldU ratios were evaluated, only ongoing forks with red-green patterns were considered. **b** DNA combing IdU to CldU track length ratios, for asynchronous parental (WT) cells and *RECQL4* KO and HD clones, from the experiment of Fig. 3d-f. ns; not significant. **c** and **d** Fork progression, as assessed by EdU-seq at the indicated time points after HU release in parental (WT) cells and *RECQL4* KO and HD clones. The data presented are from the experiment of Fig. 3g-i. NE, normal levels of Cyclin E (**b**); OE, overexpression of Cyclin E (**c**).

Supplementary Figure 5

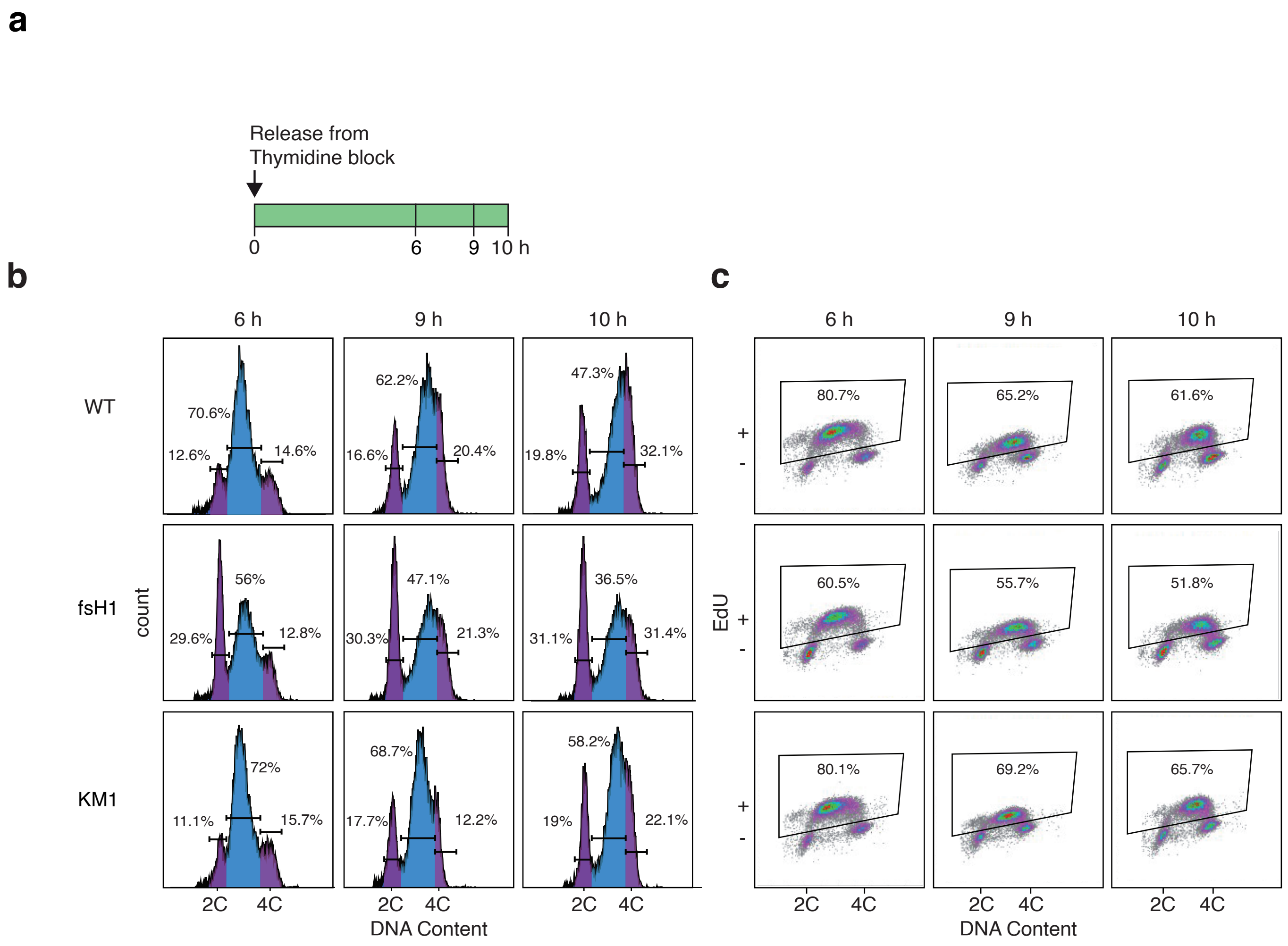

Supplementary Fig. 5 | Kinetics of S-phase progression.

a Experimental outline. Cells were synchronized in early S phase with a single thymidine block and released in fresh medium for 6, 9 or 10 hours. EdU was added to the media 30 minutes before harvesting the cells. **b** Flow cytometry histograms monitoring the genomic DNA content of parental (WT) cells and *RECQL4* mutant clones at the indicated time points. Small fractions of cells, that are irreversibly arrested in G1 or G2 by the thymidine treatment, serve as markers of 2C and 4C DNA content, respectively. **c** Flow cytometry scatter plots monitoring EdU incorporation and DNA content.

Supplementary Figure 6

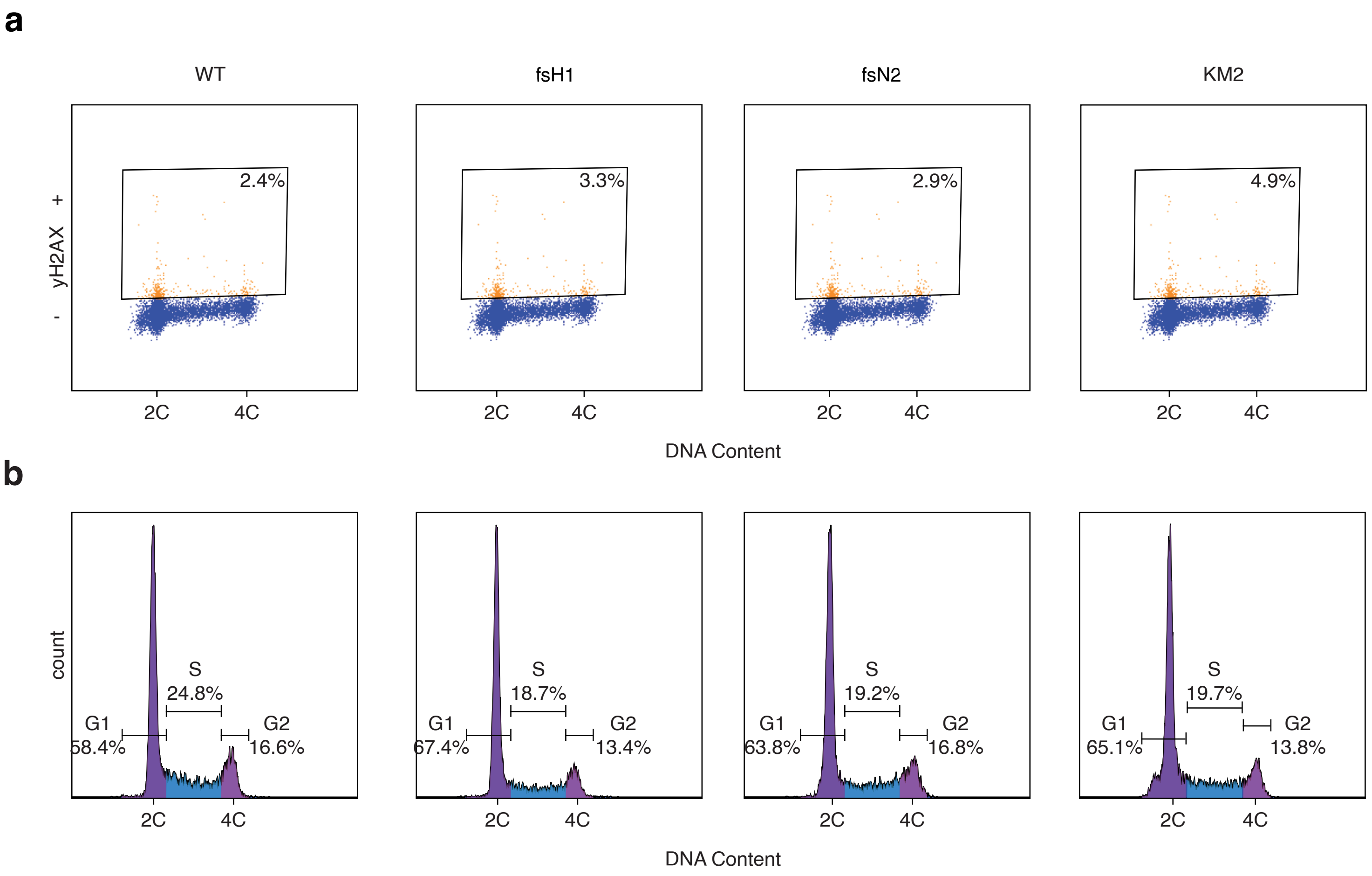

Supplementary Fig. 6 | Absence of a DNA damage response in *RECQL4* mutant clones.

a Flow cytometry scatter plots monitoring γH2AX levels and DNA content in asynchronous parental (WT) cells and *RECQL4* mutant clones expressing normal levels of Cyclin E. The gate and percentages indicate the γH2AX-positive cells. The differences in percentages between the parental and *RECQL4* mutant clones are within the errors of the experiment. **b** Flow cytometry histograms of DNA content of asynchronous parental (WT) cells and *RECQL4* mutant clones from the experiment shown in (**a**). The percentages of cells in the G1, S and G2 phases of the cell cycle are indicated.

Supplementary Table 1

Cell Line Name	Cell Line description	Sample Name	Genetic Background - Perturbations	Experimental Setup
U2OS (Tet-Off CCNE overexpression)	Human Bone Osteosarcoma Epithelial Cells	fsH1_NE_MiDASseq	KO - CRISPR frameshift mutation in the helicase domain of the RECQL4 gene	18hrs_Thymidine_Block/14.5hrs_Aphidicolin+RO3306/1.5hrs_Aphidicolin+RO3306+HU/1hr_HU_EdU_Nocodazole
		KM1_NE_MiDASseq	Substitution of K508M that inactivates helicase activity	18hrs_Thymidine_Block/14.5hrs_Aphidicolin+RO3306/1.5hrs_Aphidicolin+RO3306+HU/1hr_HU_EdU_Nocodazole
		WT_NE_MiDASseq_rep1	Wild Type cells	18hrs_Thymidine_Block/14.5hrs_Aphidicolin+RO3306/1.5hrs_Aphidicolin+RO3306+HU/1hr_HU_EdU_Nocodazole
		WT_NE_MiDASseq_rep2	Wild Type cells	18hrs_Thymidine_Block/14.5hrs_Aphidicolin+RO3306/1.5hrs_Aphidicolin+RO3306+HU/1hr_HU_EdU_Nocodazole
		KM1_OE_EdUseq_R30	Substitution of K508M that inactivates helicase activity	8hrs_Nocodazole/Mitotic_Shake-Off/14hrs_HU/Release_30min_EdU
		KM2_NE_EdUseq_R30	Substitution of K508M that inactivates helicase activity	8hrs_Nocodazole/Mitotic_Shake-Off/14hrs_HU/Release_30min_EdU
		WT_NE_EdUseq_R30	Wild Type cells	8hrs_Nocodazole/Mitotic_Shake-Off/14hrs_HU/Release_30min_EdU
		WT_OE_EdUseq_R30	Wild Type cells	8hrs_Nocodazole/Mitotic_Shake-Off/14hrs_HU/Release_30min_EdU
		KM1_OE_EdUseq_R90	Substitution of K508M that inactivates helicase activity	8hrs_Nocodazole/Mitotic_Shake-Off/14hrs_HU/Release_90min_EdU
		KM2_NE_EdUseq_R90	Substitution of K508M that inactivates helicase activity	8hrs_Nocodazole/Mitotic_Shake-Off/14hrs_HU/Release_90min_EdU
		WT_NE_EdUseq_R90_rep1	Wild Type cells	8hrs_Nocodazole/Mitotic_Shake-Off/14hrs_HU/Release_90min_EdU
		WT_NE_EdUseq_R90_rep2	Wild Type cells	8hrs_Nocodazole/Mitotic_Shake-Off/14hrs_HU/Release_90min_EdU
		WT_OE_EdUseq_R90	Wild Type cells	8hrs_Nocodazole/Mitotic_Shake-Off/14hrs_HU/Release_90min_EdU
		fsH2_NE_EdUseq	KO - CRISPR frameshift mutation in the helicase domain of the RECQL4 gene	8hrs_Nocodazole/Mitotic_Shake-Off/14hrs_HU+EdU
		fsN2_NE_EdUseq	KO - CRISPR frameshift mutation in the N-terminal domain of the RECQL4 gene	8hrs_Nocodazole/Mitotic_Shake-Off/14hrs_HU+EdU
		KM2_NE_EdUseq	Substitution of K508M that inactivates helicase activity	8hrs_Nocodazole/Mitotic_Shake-Off/14hrs_HU+EdU
		WT_NE_EdUseq	Wild Type cells	8hrs_Nocodazole/Mitotic_Shake-Off/14hrs_HU+EdU
		fsH2_OE_EdUseq	KO - CRISPR frameshift mutation in the helicase domain of the RECQL4 gene	8hrs_Nocodazole/Mitotic_Shake-Off/6hrs_HU+EdU
		fsN2_OE_EdUseq	KO - CRISPR frameshift mutation in the N-terminal domain of the RECQL4 gene	8hrs_Nocodazole/Mitotic_Shake-Off/6hrs_HU+EdU
		KM1_OE_EdUseq	Substitution of K508M that inactivates helicase activity	8hrs_Nocodazole/Mitotic_Shake-Off/6hrs_HU+EdU
		KM2_OE_EdUseq	Substitution of K508M that inactivates helicase activity	8hrs_Nocodazole/Mitotic_Shake-Off/6hrs_HU+EdU
		WT_OE_EdUseq_rep1	Wild Type cells	8hrs_Nocodazole/Mitotic_Shake-Off/6hrs_HU+EdU
		WT_OE_EdUseq_rep2	Wild Type cells	8hrs_Nocodazole/Mitotic_Shake-Off/6hrs_HU+EdU
		fsH2_NE_EdUseq_R30	KO - CRISPR frameshift mutation in the helicase domain of the RECQL4 gene	8hrs_Nocodazole/Mitotic_Shake-Off/14hrs_HU/Release_30min_EdU
		fsH2_NE_EdUseq_R90	KO - CRISPR frameshift mutation in the helicase domain of the RECQL4 gene	8hrs_Nocodazole/Mitotic_Shake-Off/14hrs_HU/Release_90min_EdU
		fsN2_NE_EdUseq_R30	KO - CRISPR frameshift mutation in the N-terminal domain of the RECQL4 gene	8hrs_Nocodazole/Mitotic_Shake-Off/14hrs_HU/Release_30min_EdU
		fsN2_NE_EdUseq_R90	KO - CRISPR frameshift mutation in the N-terminal domain of the RECQL4 gene	8hrs_Nocodazole/Mitotic_Shake-Off/14hrs_HU/Release_90min_EdU
		fsH2_OE_EdUseq_R30	KO - CRISPR frameshift mutation in the helicase domain of the RECQL4 gene	8hrs_Nocodazole/Mitotic_Shake-Off/14hrs_HU/Release_30min_EdU
		fsH2_OE_EdUseq_R90	KO - CRISPR frameshift mutation in the helicase domain of the RECQL4 gene	8hrs_Nocodazole/Mitotic_Shake-Off/14hrs_HU/Release_90min_EdU
		fsN2_OE_EdUseq_R30	KO - CRISPR frameshift mutation in the N-terminal domain of the RECQL4 gene	8hrs_Nocodazole/Mitotic_Shake-Off/14hrs_HU/Release_30min_EdU
		fsN2_OE_EdUseq_R90	KO - CRISPR frameshift mutation in the N-terminal domain of the RECQL4 gene	8hrs_Nocodazole/Mitotic_Shake-Off/14hrs_HU/Release_90min_EdU

Supplementary Table 1 | List of high throughput sequencing samples.

NE, normal levels of Cyclin E; OE, overexpression of Cyclin E; R30, release 30 minutes; R90, release 90 minutes; rep1, replicate 1; rep2, replicate 2. Raw and processed files can be found at the GEO database under accession number: GSE225532.
